# Bottlenecks and opportunities in field-based high-throughput phenotyping for heat and drought stress

**DOI:** 10.1093/jxb/erab021

**Published:** 2021-01-20

**Authors:** Nathan T Hein, Ignacio A Ciampitti, S V Krishna Jagadish

**Affiliations:** 1 Department of Agronomy, Kansas State University, Manhattan, KS, USA; 2 The University of Queensland, Australia

**Keywords:** Drought stress, field-based high-throughput phenotyping, heat stress, photosynthetic efficiency, remote sensing, time of day of flowering, water-soluble carbohydrates, yield estimation

## Abstract

Flowering and grain-filling stages are highly sensitive to heat and drought stress exposure, leading to significant loss in crop yields. Therefore, phenotyping to enhance resilience to these abiotic stresses is critical for sustaining genetic gains in crop improvement programs. However, traditional methods for screening traits related to these stresses are slow, laborious, and often expensive. Remote sensing provides opportunities to introduce low-cost, less biased, high-throughput phenotyping methods to capture large genetic diversity to facilitate enhancement of stress resilience in crops. This review focuses on four key physiological traits and processes that are critical in understanding crop responses to drought and heat stress during reproductive and grain-filling periods. Specifically, these traits include: (i) time of day of flowering, to escape these stresses during flowering; (ii) optimizing photosynthetic efficiency; (iii) storage and translocation of water-soluble carbohydrates; and (iv) yield and yield components to provide in-season yield estimates. Moreover, we provide an overview of current advances in remote sensing in capturing these traits, and discuss the limitations with existing technology as well as future direction of research to develop high-throughput phenotyping approaches. In the future, phenotyping these complex traits will require sensor advancement, high-quality imagery combined with machine learning methods, and efforts in transdisciplinary science to foster integration across disciplines.

## Teaser

This review focuses on developing high-throughput phenotyping approaches to quantify key physiological traits at high temporal frequency, involving diverse germplasm to incorporate greater heat and drought stress resilience in crops.

## Introduction

Advancements in quantifying the impact of abiotic stress on the productivity of field crops have become more important than ever in order to breed for heat and drought stress resilience or to understand the ability of a plant to maintain yield under stressful environments. In order to meet the future food demand, global agriculture production must be doubled by 2050 as compared with 2012 (Food and Agriculture Organization, 2017, 2019). As of 2008, it has been shown that yields in major crops such as maize (*Zea mays* L.), rice (*Oryza sativa* L.), and wheat (*Triticum aestivum* L.) are increasing at an annual rate of 1.6, 1.0, and 0.9%, respectively (Ray *et al.*, 2013). If the same rate of increase is sustained, maize, rice, and wheat would see an increase in production of 67, 42, and 38%, respectively, by 2050. This rate of increase, obtained largely through advances in breeding aided by high technology management, has mitigated the negative effects due to a challenging and damaging climate until now, but, as demand grows and climate instability continues to increase, these negative effects could pose a threat to global food security in the future.

The Intergovernmental Panel on Climate Change (IPCC) has predicted that heat waves in the future will occur at a more frequent rate and with increases in both duration and intensity (IPCC, 2014). The increase in global mean temperature and the expected instability in precipitation creates a potential major risk to global food security. Gourdji *et al.* (2013) have predicted that, by 2030, 31% of maize-, 16% of rice-, and 11% of wheat-growing areas will record over five reproductive days with temperatures above their respective critical threshold, in any given year. This increase in temperature coinciding with sensitive developmental stages, such as flowering, will have detrimental impacts on yield (Jagadish, 2020). Empirically, heat stress during the booting and flowering stages in rice reduced yield by as much as 28.5% depending on the timing and duration of heat stress (Aghamolki *et al.*, 2013). Similarly, a significant reduction in winter wheat yield was recorded with heat stress coinciding with heading and lasting for 15 d, even though a stress period of 5 d was sufficient to induce yield loss (Balla *et al.*, 2019). In addition, it is predicted that with every degree centigrade increase in mean temperature, the global wheat production will be reduced by 6% (Asseng *et al.*, 2015).

Drought reduced yield in ~75% of all globally harvested areas of maize, rice, wheat, and soybeans (*Glycine max* L.) between 1983 and 2009 (Kim *et al.*, 2019). The IPCC has also predicted a shift in the water cycle where the higher latitudes will receive increased precipitation while the mid-latitudes and those areas already prone to drought will encounter a more substantial decrease in water supply (IPCC, 2014). Daryanto *et al.* (2016) synthesized 144 studies between 1980 and 2015, and reported an average yield reduction of ~21% for wheat and 39% for maize due to drought. Zhang *et al.* (2018), using a meta-analysis approach including >110 independent studies, recorded a 28% and 25% yield reduction due to drought in wheat and rice, respectively, with the largest reduction associated with stress during grain filling. Similarly, Sehgal *et al.* (2018) reported that the most critical growth stages with significant reductions in yield due to drought and/or heat stress were the reproductive and grain-filling stages. Hence, a better understanding of a plant’s responses to both heat and drought stresses during reproductive and grain-filling stages is crucial to provide new opportunities for breeding programs to enhance the rate of success in developing stress-tolerant genotypes.

Remote sensing approaches allow for data collection on much larger studies encompassing a wide genetic diversity in order to phenotype for abiotic stress resilience. Remote sensing has been utilized for a variety of purposes such as measuring canopy height (Varela *et al.*, 2017; Thompson *et al*., 2018, 2020; Zhou *et al.*, 2020), biomass (Neumann *et al.*, 2015; Padilla-Chacón *et al.*, 2019), canopy temperature (Romano *et al.*, 2011; Pauli *et al.*, 2016; Graß *et al.*, 2020), and leaf area (Neilson *et al.*, 2015; Zhang *et al.*, 2019), and predicting yield (Rischbeck *et al.*, 2016; Becker and Schmidhalter, 2017; El-Hendawy *et al.*, 2017; Zhou *et al.*, 2020). Through the use of specialized vegetation indices (VIs) or spectral bands alone, remote sensing can quickly and efficiently collect data on different traits simultaneously, non-destructively, and with a high spatio-temporal frequency. In addition, remote sensing presents the opportunity for correlating an index with the trait of interest, without being confounded by a differential time-stamp, unlike manual measurements (Janse, 2017; Xue and Su, 2017).

In order to effectively utilize remote sensing for the diagnosis of drought and heat stress impacts on crops, the data obtained should help in understanding complex physiological processes that determine yield, at a scale that cannot be achieved by manual methods. Recently, there have been attempts to review advances in sensor technology and estimation of agronomic traits such as plant height, biomass, or greenness (Araus *et al.*, 2018; Chawade *et al.*, 2019). Hence, to avoid duplication, this review utilizes the progress achieved in the realm of sensor technology and focuses on quantifying key physiological traits or processes that are critical in understanding crop resilience to drought and heat stress during the reproductive period, but more specifically with focus on the grain-filling period. These specific traits include phenotyping for (i) time of day of flowering (TOF), as a means to escape heat stress during flowering; (ii) enhancement of photosynthetic efficiency by optimizing stay-green versus senescence; (iii) translocation of water-soluble carbohydrates (WSCs) and their contribution to yield under stress; and (iv) yield components (i.e. grain number and grain size determination) to provide in-season (and before harvest) yield estimates. These traits define major physiological and agronomic aspects related to heat and drought stress resilience in crops and are complex, labor-intensive, time-consuming to measure, and change dynamically over time so that they cannot be effectively captured through traditional methods. Opportunities exist with each trait to increase the throughput and accuracy of trait determination via remote sensing. This review aims to identify ways to utilize advances in remote sensing and to strengthen efforts towards developing heat- and drought-tolerant crops for the future. Finally, the review identifies limitations and bottlenecks in remote sensing methods and provides recommendations for future research in order to overcome these limitations.

## Time of day of flowering: a route to escape heat stress

Historically, adaption to abiotic stresses has been acquired naturally in crops through evolution, but crops are not equipped to deal with significant intra- and interannual climate variability faced under current and predicted future climate. Traits that induce heat stress resilience can be classified into three categories: tolerance, avoidance, and escape. Tolerance is defined by the ability of the plants to continue operating their physiological processes under stressful conditions (Khan *et al.*, 2014). Traits that define avoidance allow normal processes to continue by creating a more favorable microclimate. An excellent example of heat stress avoidance is transpirational cooling, wherein canopy temperature is decreased to optimal levels even under severe ambient hotter environments (Lin *et al.*, 2017). This trait, however, is highly beneficial under sufficient water supply (Julia and Dingkuhn, 2013), but not favorable under combined drought and heat stresses, as the competition to conserve water to survive drought is prioritized (Lin *et al.*, 2017). Escape, on the other hand, provides the opportunity for sensitive physiological processes to occur during favorable times of the season (macro-escape) or the day (micro-escape).

**Fig. 1.**
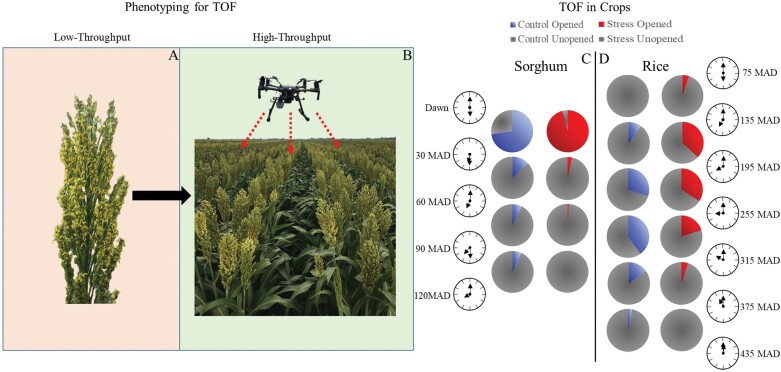
Quantifying time of day of flowering (TOF) in crops. The figure shows potential transition of methodologies in recording TOF in crops and provides case studies related to TOF in sorghum and rice. (A) Traditional low-throughput measurement via manual counts which is labor intensive, induces temporal variability, and is subject to human error. (B) Use of low-altitude UAVs and high-resolution imagery to easily acquire high-temporal and accurate data to record TOF. Natural alteration of flowering time (MAD, minutes after dawn) in sorghum (C; Chiluwal *et al.*, 2020) and the change in flower opening time in rice by genetic incorporation of early morning flowering trait (see far right pie charts) from wild rice into a popular variety (D; Ishimaru *et al.*, 2010).

Shortening crop growth duration in order to complete their life cycle or to prevent exposure to severe hot and dry summers would be an example of macro-escape (Stone, 2001; Barnabás *et al.*, 2008), while adjusting their sensitive flowering time to cooler hours with more favorable vapor pressure deficit (VPD) conditions is an example of micro-escape (Sheehy *et al.*, 2005; Jagadish, 2020). Heat stress and higher VPD during flowering lead to significant yield reductions in a large variety of crops, and the inclusion (naturally or through genetic improvement) of an early morning flowering trait has been shown to significantly reduce spikelet sterility and yield losses in rice (Ishimaru *et al.*, 2010; Hirabayashi *et al.*, 2015; Bheemanahalli *et al.*, 2017) and sorghum (Chiluwal *et al.*, 2020). Although crops can employ tolerance, avoidance, or escape independently or in combination, this section focuses on advancing methods to phenotype for TOF as an effective means to minimize crop damage from heat and drought stresses (Jung and Müller, 2009; Jagadish *et al.*, 2015). Currently, the TOF is manually phenotyped, which is tedious, prone to human error (liable to bias), confounded by spatio-temporal variability of measurements, and can only be measured on a limited number of genotypes (Ishimaru *et al.*, 2010; Aiqing *et al.*, 2018; Chiluwal *et al.*, 2020; Pokharel *et al.*, 2020) (Fig. 1A). Traditionally, researchers have identified the flowering pattern in crops by counting the number of opened flowers at specific time increments or by marking opened flowers by fine-tipped pens (Hirabayashi *et al.*, 2015), but this can lead to confounding results as any physical stimuli can alter the flowering pattern (Kobayasi *et al.*, 2010).

Steps toward optimizing this methodology to reduce temporal variability with manual measurements and to overcome physical stimuli induced by human touch have been proposed. For rice, Kobayasi *et al.* (2010) utilized digital cameras to determine the flower opening time. This allowed for more frequent measurements (10 min intervals), and created a physical representation of the inflorescence at the specified time point so it could be evaluated, repeatedly if necessary, at a later date. This approach also enhanced accuracy by utilizing a tripod and a timer to initiate the data collection. Significant steps have been made in the last few years, which have allowed the number of genotypes phenotyped to be increased, and reduced the variability in measurements. The first step forward came via utilizing fixed field-based phenotyping systems such as the Field Scanalyzer phenotyping platform. The unit contains multiple sensors including high-resolution digital cameras which, when combined with machine learning, can positively identify flowering in wheat (Sadeghi-Tehran *et al.*, 2017). This methodology has an accuracy ranging from 76% to 92%, and its imprecisions are linked to the size and color of the anthers as they can vary amongst genotypes (Sadeghi-Tehran *et al.*, 2017) (Table 1). A significantly greater challenge in determining the flower opening time was observed in *Setaria viridis*, wherein the flower opening was predominant during the night in all three tested accessions (Desai *et al.*, 2018). A Raspberry Pi system equipped with infrared imaging allowed the authors to correlate the flower opening time in the night with the movement in floral bristles, coinciding with extrusion of anthers (Desai *et al.*, 2018) (Table 1). The ability to capture the night-time flower opening is important to quantify the trait in some wild species known to predominantly flower during the night (rice; Sheehy *et al.*, 2007) or for capturing late evening flowering as seen in wheat (Aiqing *et al.*, 2018).

**Table 1.** Overview of research advances for phenotyping target traits focused on in the review

Trait	Crop	Throughput	Location	Platform	Sensor	Sensor-measured trait	Observed agronomic trait	Reference
Time of day of flowering	Wheat	High	Field	Field scanalyzer	RGB digital camera	TOF	TOF	Sadeghi-Tehran *et al.* (2017)
	*Setaria viridis*	Medium	Lab	Fixed mount	RGB digital camera (daytime)	TOF	TOF	Desai *et al.* (2018)
					Infrared camera (night-time)	TOF	TOF	
	Wheat	High	Field	Tractor mount	RGB digital camera	Percent heading	Percent heading	Wang *et al.* (2019)
Photosynthetic efficiency	Barley and sugar beet	Medium	Field	Fixed mount	LIFT system	Chl *a*	Daily average fluorescence values	Raesch *et al.* (2014)
	Aspen and cherry tree	Medium	Field/lab	Handheld	Hyperspectral radiometer	NDRE_740_	Photosynthetic efficiency	Peng *et al.* (2017)
					SPAD meter	Chlorophyll index	Photosynthetic efficiency	
	Evergreen shrub	Medium	Field	Handheld	Field spectroradiometer	PRI	Photosynthetic efficiency	Zhang *et al.* (2017)
							Yield	
Translocation of WSCs	Wheat	High	Field	Tractor mount	Hyperspectral radiometer	Spectral region (350–1290 nm)	WSC amount	Dreccer *et al.* (2014)
	Maize	Medium	Field	Handheld	Hyperspectral radiometer	Reflectance spectra	Sucrose content	Yendrek *et al.* (2017)
	Wheat	Medium	Field	Fixed mount	Hyperspectral radiometer	Spectral region (350–2500 nm)	WSC concentration	Garriga *et al.* (2017)
Estimating yield and yield parameters	Rice	Medium	Field	Fixed mount	RGB digital camera	Panicle count	Panicle count	Xiong *et al.* (2017)
	Wheat	Medium	Field	Tractor mount	RGB digital camera	Spike count	Spike count	Hasan *et al.*, 2018
	Wheat	Medium	Field	Handheld	RGB digital camera	Ear count	Ear density	Madec *et al.* (2019)
	Sorghum	High	Field	UAV-based	RGB digital camera	Head count	Head count	Guo *et al.* (2018)
	Sorghum	High	Field	UAV-based	RGB digital camera	Head count	Head count	Ghosal *et al.* (2019)
	Sorghum	High	Field	UAV-based	RGB digital camera	Head count	Head count	Lin and Guo (2020)

A mobile methodology has been developed by utilizing a high-clearance field-based high-throughput mobile phenotyping platform outfitted with multiple high-resolution digital cameras which collected geo-referenced images with the help of a built-in real-time kinematic global positioning system (RTK GPS) (Barker *et al.*, 2016; Wang *et al.*, 2019). Utilizing deep learning tools, this methodology is able to correctly identify plant phenology and growth stages, and the system was utilized to identify flowering dates, which were associated with plot-based breeder’s score (Wang *et al.*, 2019) (Table 1). The system, however, was not employed to identify TOF due to lack of high temporal measurements on a single day. The success of the system in identifying heading and flowering dates indicates that the system is sensitive enough to be modified to capture images at a high temporal setting to explore the flowering pattern in different crops.

The success of utilizing both fixed field-based phenotyping systems and ground-based mobile phenotyping platforms indicates that aerial high-throughput phenotyping for capturing TOF in crops is achievable. Unmanned aerial vehicles (UAVs) are capable of carrying extremely high-resolution red–green–blue (RGB) digital cameras and, as cameras have become smaller, this has allowed even smaller UAVs to carry them (Colomina and Molina, 2014) (Fig. 1B). Low-altitude flights will allow the capture of extremely high-resolution images of the canopy in order to quantify the TOF. Two examples for the TOF phenomenon are presented wherein sorghum and rice genotypes vary in the proportion of flowers that open at different times of the day (Fig. 1C, D). The extremely short window (minutes after dawn) in sorghum and a much longer flowering window (hours after dawn) in rice provides the diversity in the scale of operation in crops with TOF, and the efficiency and accuracy required to capture the genetic diversity for this trait. The difference in color between the green leaf background with a contrasting yellow of the anthers provides the opportunity to establish a phenotyping approach that can employ an area- and color-based detection method to define the temporal magnitude of flowering (Fig. 1C, D). Employing this method will allow for screening a large number of genotypes, at high spatio-temporal frequency, and with increased effectiveness, thereby facilitating integration of this trait into abiotic stress breeding programs.

## Photosynthetic efficiency: capturing stay-green versus senescence dynamics

Photosynthesis is one of the key physiological processes which can be optimized for the achievement of maximum yield potential in crops, with abiotic stresses negatively impacting photosynthetic efficiency which can significantly reduce grain yields (Crafts-Brandner and Salvucci, 2002; Long *et al.*, 2006; Feng *et al.*, 2013; Ambavaram *et al.*, 2014). Attainable maximum yield can be determined by analyzing the amount of light captured, the ability of the plant to convert this energy into biomass, and the proportion of biomass partitioned to grain (Muchow *et al.*, 1990). Improvements in the amount of radiation captured and increases in the partitioning of biomass into grain have been achieved through plant breeding; however, there is room for further improvement in efficiency in translating intercepted radiation into biomass. Theoretically, maximum potential photosynthetic efficiencies are indicated to be 0.051 for C_3_ and 0.060 for C_4_ crops (Long *et al.*, 2005). The maximum short-term rates of photosynthetic efficiency recorded reached ~70% of this potential in both C_3_ and C_4_ plants, while the maximum season-long measured efficiencies were ~47% of the maximum for C_3_ and 57% of the maximum for C_4_ crops (Monteith, 1977; Beadle and Long, 1985; Piedade *et al.*, 1991; Beale and Long, 1995). Thus, increasing yields to meet the future global demand will rely on the further improvement of photosynthetic efficiency or the ability of crops to convert captured light energy into biomass.

Possible developments to improve photosynthetic efficiency for heat and drought stress resilience include introducing the C_4_ photosynthetic pathway into C_3_ plants, improving Rubisco kinetic properties, and increasing photoprotection to reduce high levels of reactive oxygen species (Gowik and Westhoff, 2011; Murchie and Niyogi, 2011; Whitney *et al.*, 2011). Heat and drought stress can increase the oxygenation reaction of Rubisco, which can result in a direct loss of up to 30% of fixed carbon (Raines, 2011). This degradation of fixed carbon is extremely influential on potential yield when drought or heat stress occur during flowering or grain filling. In addition, the early onset of senescence due to abiotic stresses is characterized by accelerated chlorophyll degradation and severely reduced photosynthetic efficiency (Hörtensteiner and Feller, 2002; Woo *et al.*, 2018). These negative effects can be reduced through functional stay-green phenotypes, by extending the activity of the photosynthetic machinery (Thomas and Ougham, 2014). Functional stay-green phenotypes are shown to have a positive effect on either yield, heat, or drought stress tolerance in sorghum (*Sorghum bicolor* L.) (Borrell *et al.*, 2014), wheat (Spano *et al.*, 2003; Pinto *et al.*, 2016), barley (*Hordeum vulgare* L.) (Seiler *et al.*, 2014; Gous *et al.*, 2015), maize (Cairns *et al.*, 2012), and rice (Fu *et al.*, 2011).

Traditional measurements of photosynthetic efficiency are laborious, destructive, and fail to detect the subtle changes that occur at the inception of senescence (Šebela *et al.*, 2020). Sequential biomass harvests have been proposed to capture the photosynthetic efficiency for the entire growing season (Zhu *et al.*, 2010), which is highly cumbersome to achieve with large breeding populations. A major milestone in addressing the above limitation was reached through the creation of the laser-induced fluorescence transient (LIFT) method for remotely measuring this plant trait (Raesch *et al.*, 2014) (Table 1). The LIFT technique uses a laser at 665 nm to excite the leaves, and the fluorescent emission at 690 nm by the plant is collected by a reflective telescope and processed (Kolber *et al.*, 2005; Pieruschka *et al.*, 2012). Advancements have been made in the mobility of this system to be utilized with highly precise GPS in a field setting; however, it is still quite bulky and requires a large cart or all-terrain vehicle for its operation (Muller *et al.*, 2018). Another limitation of the system is that it can measure an area larger than the targeted leaf, which can confound conclusions due to overlap of multiple layers of leaves within the canopy (Raesch *et al.*, 2014).

A study using hyperspectral imaging on evergreen tree leaves exposed to a simulated short-term drought stress revealed a reduction in photosynthetic efficiency well before chlorophyll degradation was initiated. The use of longwave red-edge vegetation indices such as the red-edge normalized difference vegetation index (NDVI; NDRE740) and red-edge chlorophyll index (CI740) had significantly strong relationship with photosynthetic efficiency (*R*^2^=0.88 and 0.72 for stressed and non-stressed leaves, respectively) (Peng *et al.*, 2017) (Table 1). The photochemical reflectance index (PRI) has a similar strong relationship with photosynthetic efficiency in flowering plant species under control, drought, and warming scenarios (*R*^2^=0.78–0.85) (Zhang *et al.*, 2017) (Table 1).

**Fig. 2.**
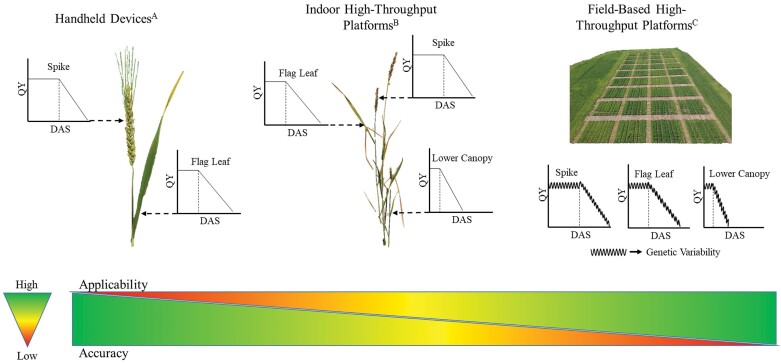
Optimizing stay-green and senescence dynamics. Handheld, indoor high-throughput, and field-based high-throughput techniques for quantifying photosynthetic efficiency are presented using effective quantum yield of PSII (QY) as a case study. Handheld devices (A), though sensitive enough to detect subtle changes such as initiation of senescence, are highly laborious, provide data either at a leaf or spike level, and are challenging to be deployed for large-scale phenotyping. Indoor high-throughput platforms (B) having similar or higher sensing capability can easily acquire trait information on the whole plant automatically without human intervention. Field-based high-throughput platforms (C) have the capability of gathering reflectance data on a large number of genotypes with extreme sensitivity and low temporal variation.

The chlorophyll fluorescence, which is shown to quantify photosynthetic efficiency, has been used to measure the effective quantum yield (QY) of PSII in order to determine the exact change point at which senescence begins in leaves and floral tissue (Šebela *et al.*, 2015, 2020). Chlorophyll fluorescence measured through QY provides information on the overall efficiency of photochemical reactions in PSII under the light-adapted state (Genty *et al.*, 1989), and has been effectively utilized to phenotype a rice diversity panel exposed to water-deficit stress (Šebela *et al.*, 2019). Therefore, using QY as a case study trait, the transition from leaf (handheld) to the plot level using UAVs and the desired phenotype for stress-prone environments with source–sink-related stay-green and senescence patterns is presented in Fig. 2. The UAV platforms provide the opportunity to move beyond point-based leaf or inflorescence-based photosynthetic parameter measurements (Šebela *et al.*, 2015, 2020; Fig. 2A) to whole plant- (Fig. 2B) or canopy-based estimations (Fig. 2C), to capture genetic diversity for extending source–sink photosynthetic efficiency. Developing varieties that can trigger senescence in the lower half of the plant or plot while retaining active photosynthetic machinery in the top half or third is a desirable phenotype for heat and drought stress-prone environments (Jagadish *et al.*, 2015). This ideotype concept proposed can be realized using advances in the sensor-based technology to help capture the differential onset and rate of senescence at different positions along the plant or plot in large diversity panels or mapping populations (Fig. 2C). Photosynthetic efficiency is an integrated measure of many physiological processes which are difficult to be determined individually through experiments, hence requiring a modeling framework to design a phenotype that can optimize both resource capture and use efficiency to increase yield. Determining opportunities to further enhance photosynthetic efficiency is an ideal target for designing an ideotype through model-based approaches (Hammer *et al.*, 2010; Lobell *et al*., 2013, 2014; Y. Wu *et al.*, 2019). These approaches can help breed for varieties optimized with functional stay-green versus senescence, and enhance assimilate production and transport efficiency to sustain productivity under heat- and drought-prone environments.

## Translocation of water-soluble carbohydrates

The end result of photosynthesis is the production of monosaccharides such as glucose and fructose, which form the foundation blocks for storage carbohydrates (polysaccharides) such as starch. Sugars including glucose and fructans, synthesized in leaves, are transported to the stem and leaf sheaths and stored as WSCs (also known as non-structural carbohydrates) (Schnyder, 1993; Gebbing, 2003; Ehdaie *et al.*, 2006; Fernandez *et al.*, 2020). Subsequently after storage, the accrued WSCs in the stem and leaf sheaths are remobilized to the sink tissue during grain filling (Scofield *et al.*, 2009), with the efficiency of translocation influenced by the genetic diversity in sink strength depending on the crop or species (Cock and Yoshida 1971; Yoshida, 1981; Kiniry *et al.*, 1992; Kiniry, 1993; Schnyder, 1993; Li *et al.*, 2017).

After removing maintenance costs which can account for up to 68% of total WSC allocation, in wheat as much as 0.68–0.78 g of yield can be produced for each 1 g of WSCs stored through apparent reserve use (Kiniry, 1993). An increased rate of reallocation due to terminal drought has been stated to contribute up to 50% of yield in traditional—and as much as 70% in elite—cultivars (van Herwaarden *et al.*, 1998). Similar responses have been reported with heat (Schittenhelm *et al.*, 2020) and other biotic stresses (Sadras *et al.*, 2020). To minimize damage from stresses, newer phenotyping methods for high WSC storage and translocation are recommended in crops (Blum, 1998; Asseng and van Herwaarden, 2003; Wang *et al.*, 2016; Schittenhelm *et al.*, 2020). Studies exploring genotypic variation for WSC levels have been mainly focused on barley (Gay *et al.*, 1999), wheat (van Herwaarden *et al.*, 2003; Ruuska *et al.*, 2006; Dreccer *et al.*, 2009; Ovenden *et al.*, 2017), rice (Xiong *et al.*, 2014; Wang *et al*., 2016, 2017; Moura *et al.*, 2017), and maize (Jones and Simmons, 1983; Uhart and Andrade, 1995; Edreira *et al.*, 2014; A. Wu *et al.*, 2019; Fernandez *et al.*, 2020).

Traditional methodology for quantifying WSC levels is destructive, time-consuming, expensive, and restricts the number of genotypes or samples that can be realistically processed. Due to the time-consuming nature of sample gathering and processing for WSCs, temporal changes can occur within plant samples in response to the changes in the prevailing microclimate. This indicates the need for a high-throughput methodology which can quickly and accurately measure WSC levels in a large number of samples. Currently, lab-based methods for the extraction of WSCs utilize different approaches in wet chemistry. The original method was developed in 1954 by using anthrone and is still used to this day for ground-truthing or for generating benchmarks or references (Yemm and Willis, 1954; Giri, 2019). To increase throughput, near-infrared reflectance spectroscopy (NIRS) is being utilized alongside traditional wet chemistry methods. This medium-throughput methodology begins by determining the WSC levels in a subset of samples via wet chemistry and then the data generated are correlated with NIRS reflectance spectra. This methodology has been utilized on different crops including wheat (Rebetzke *et al.*, 2008; Wang *et al.*, 2011; Giri, 2019) rice (Wang *et al.*, 2016), and maize (Campo *et al.*, 2013).

The first step towards a true high-throughput phenotyping method for stem WSC levels was attempted on four recombinant inbred wheat lines utilizing a hyperspectral radiometer (Dreccer *et al.*, 2014). The radiometer, with a sampling range from 350 nm to 2500 nm, was mounted onto a four-wheel drive motorbike at 1.35 m above the soil. The remotely sensed WSC levels were then confirmed in the laboratory utilizing the anthrone method, presenting a significantly strong relationship (*R*^2^=0.90) averaged across 2 years (Dreccer *et al.*, 2014) (Table 1). It was not until 2017 that hyperspectral imaging was used again to evaluate the concentration of WSCs. A study involving estimation of sucrose content in maize leaves had success in utilizing hyperspectral imaging of the adaxial surface of a leaf with an illuminated leaf clip contact probe and partial least squares regression (PLSR) models (Yendrek *et al.*, 2017) (Table 1). While not as successful as Dreccer *et al.* (2014), the PLSR model was still able to predict sucrose content within the leaf with an *R*^2^ of 0.62. Garriga *et al.* (2017) utilized the same hyperspectral radiometer model as Dreccer *et al.* (2014) to predict WSCs in a large variety trial, including 384 cultivars and advanced lines of spring wheat in both well-watered and water-stressed environments. The radiometer was placed at a 45° angle and swept over the plot three times and, utilizing multivariate regression models, the study was able to predict stem WSCs with an *R*^2^ of 0.56 (Garriga *et al.*, 2017) (Table 1). It is unclear whether the difference in coefficients of determinations between Dreccer *et al.* (2014) and Garriga *et al.* (2017) was due to the angle at which the reflectance was obtained or other confounding factors, but these procedures need to be further standardized to accurately reflect the ground-truth observational data.

The next step forward in quantifying WSC levels via a high-throughput methodology is by implementing machine learning. This methodology has not been implemented with a row crop; however, it was recently tested with perennial ryegrass. The authors used a hyperspectral radiometer as well as a light shield in order to capture the spectra under stable light conditions from 960 different plants, comprised of 50 experimental perennial ryegrass varieties (Smith *et al.*, 2020). The light shield was manually placed on each plant, and artificial light within the shield was used as the light source. Comparatively, the cubist model resulted in an *R*^2^ of 0.49 while the PLSR model was only able to obtain an *R*^2^ of 0.19 (Smith *et al.*, 2020). Although promising, the methodology may be too laborious and not be practical to implement on multilocation trials that involve diversity panels or mapping populations in order to make it applicable to breeding programs. With limited research into the feasibility of utilizing hyperspectral imaging and machine learning for rapid, accurate measurements, the methodology cannot be discredited or confirmed as the path forward for accurate high-throughput evaluation.

## Estimating yield and key yield-related parameters

The economic yield of a crop is defined as the biological yield multiplied by the harvest index (HI) of dry matter or the product of grain number and grain weight (Osaki *et al.*, 1994). The ability to accurately predict yield in both stressed and non-stressed environments is an endeavor that has been ongoing for decades. Yield prediction is a complicated undertaking due to the dynamic environmental changes that fluctuate on a large temporal scale, from daily to yearly, and on a large geographical area, from local to regional scales, resulting in large variations in attainable crop yields. This is particularly true for heat- and drought-prone environments, which lead to lower seed numbers when stress occurs immediately before or coincides with flowering (Fischer, 1985; Jagadish *et al.*, 2010; Prasad *et al*., 2015, 2017; Bheemanahalli *et al.*, 2019) or loss in seed weight with stress during grain filling (Asana *et al.*, 1958; Wardlaw, 1970, 1971; Lawas *et al.*, 2018; Hein *et al.*, 2020).

Yield forecasts for regional, national, and international cropping systems involve highly intricate and complicated systems utilizing an enormous amount of data and multiple regression models or machine learning (Jeong *et al.*, 2016; Iizumi *et al.*, 2018; Han *et al.*, 2020; Schwalbert *et al.*, 2020). Advances are being made in order to estimate yield within the season in order to aid in making important management decisions in normal, heat stress, or drought stress environments. Hence, to predict yield more reliably and accurately, particularly in abiotic stress-prone environments, approaches to remotely determine the number of heads in a plot and the number of seeds on the head are required.

The first step towards gaining the ability to predict yield is acquiring the capacity to accurately identify heads or panicles in crops. This area of remote sensing has garnered increased interest and utilizes different strategies employing machine and deep learning tools to ascertain accurate counts. One such experiment in 2017 attempted to identify rice panicles by applying a convolutional neural network (CNN) classification and entropy rate super-pixel optimization to 684 images of pot-grown rice (Xiong *et al.*, 2017). This method outperformed three previously identified methods, with an F-measure indicator, which accounts for precision and recall, of 0.77, while the previous methodologies could only reach an F-measure indicator of 0.44 (Xiong *et al.*, 2017) (Table 1).

The CNNs have also been utilized to detect and count the number of wheat spikes within a plot. This was achieved by employing a ground-based steel cart, with a central overhead rail equipped with high-resolution cameras capable of being mounted at differing angles in relation to the crop of interest (Hasan *et al.*, 2018). The Faster R-CNN model using 305 training images at different growth stages was able to attain on average a 93% accuracy on the 30 test images after training (Ren *et al.*, 2017; Hasan *et al.*, 2018) (Table 1). Similarly, another study using the same approach was equally accurate during early stages after heading, but was more robust during later stages when the leaves senesced and contrasted with greener wheat spikes (Madec *et al.*, 2019). The model achieved a high relationship (*R*^2^=0.91) when the resolution of the image was 0.26 mm but was reduced (*R*^2^=0.33) when the image resolution was decreased to 0.78 mm, indicating the need for high-resolution imagery for accurate spike detection (Madec *et al.*, 2019) (Table 1).

These advances in agricultural object identification are impressive, given how small wheat spikes are compared with other crops. Sorghum has had considerably more research into developing accurate models for extracting and counting heads (Ghosal *et al.*, 2019; Malambo *et al.*, 2019; Oh *et al.*, 2019, Preprint; Lin and Guo, 2020). Even though sorghum has a much larger sized head than wheat spikes, research faces the same challenges while utilizing UAVs to obtain imagery: changing light conditions over the duration of a flight, complex and intricate backgrounds, genotypic variations in head color, size, or shape, and overlapping heads (Guo *et al.*, 2018) (Table 1). The same authors employed a pixel-based segmentation approach to train a digital terrain surface model (DTSM) which is a supervised machine learning based on the decision tree, resulting in an F-measure of 0.92 for 52 images and 0.89 for 40 images per plot. The research group was then able to establish a deep learning framework with minimum supervision using CNN for sorghum head detection, and achieved an *R*^2^ of 0.88 with the training set comprising only 40 randomly selected images (Ghosal *et al.*, 2019) (Table 1). In a more recent study, CNN models with image segmentation accurately estimated the number of sorghum heads (*R*^2^=0.90) and characterized the shape and size of individual heads (Lin and Guo, 2020) (Table 1). This advancement could be key to estimating yield in sorghum, but seed number and weight are additional traits to be determined for effective yield prediction.

Research into using remote sensing to quantify seed number and grain weight of a plant in a field environment is limited. There has been success in controlled environments in which a 3D reconstruction of rice showed that seed number for the panicle had a significant (*P*<0.05) positive correlation with the voxel count of the reconstruction throughout the grain-filling period (*r*=0.61–0.70) (Sandhu *et al.*, 2019). This same experiment also found a significant (*P*<0.05) positive correlation (*r*=0.48–0.74) between voxel count and seed weight which increased approaching maturity. This method of obtaining the estimated seed number and weight works moderately well in the laboratory setting but will be challenging to adopt under field conditions.

**Fig. 3.**
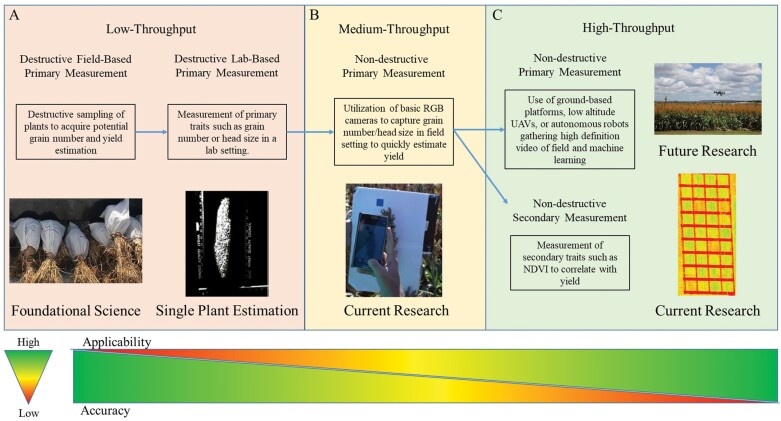
Estimation of yield and yield-related parameters. The figure illustrates the progression from destructive field-based primary measurements in order to obtain an estimation of yield to new high-throughput measurements to estimate yield through both primary and secondary measurements. The methods of gathering information for yield estimation are ordered from least applicable but highly accurate to most applicable but less accurate, or from low-throughput to high-throughput, and include destructive sampling and lab-based primary measurements (A), field-based primary measurements (B), and current investigation on developing high-throughput non-destructive primary and secondary measurements (C).

This challenge has been approached using a simpler method in order to allow for the methodology and the tool developed to be utilized by both researchers and farmers. This method follows an allometric determination method by taking RGB images with a digital camera of >1000 sorghum heads (closed panicle type). The head volume is determined by using the head length and diameter (measured using a ruler) and assuming the head is cylindrical, and comparing this volume with grain number per head resulted in a strong relationship (*R*^2^ of 0.68 and 0.58) for commercial hybrids and inbreds, respectively (Ciampitti *et al.*, 2014) (Fig. 3A). This approach has been extended to estimate final yield using variables such as row spacing and estimated seed number per kilogram of grain harvested (Ciampitti *et al.*, 2015) (Fig. 3B). The progress achieved using this method integrated with machine learning tools is currently under development (Fig. 3C). At present, the method is under substantial refinement to consider a new machine learning approach via utilization of edge detection technology for clearly defining head volume accounting for different sizes and types of panicles, namely open versus closed heads. Alternatively, current high-throughput estimations of yield are derived through the analysis of vegetation indices. While this method can provide relatively accurate prediction of yield, it is a secondary measure of yield, and the reliability of the prediction only increases near maturity and could vary based on environmental changes (Galli *et al.*, 2020). In the near future, the primary measurement based on remote sensing that is being developed (Fig. 3C) can be scalable to identify grain number on large populations in sorghum under field conditions.

## Limitations and future research directions

Keeping in line with the scope of the review, we have indicated limitations and provided recommendations for utilizing advances in sensor technology to develop high-throughput phenotyping approaches to capture physiological aspects that will help enhance heat and drought stress resilience in crops.

### Time of day of flowering

Achieving multiple flights at short temporal frequency to record TOF can be a limiting factor for many research programs. Hence, it is recommended to optimize flights that capture a large proportion of the variation on a flowering day to make the approach of using UAVs for capturing TOF feasible. In addition, the distance between the aerial sensor platform and the flowering field (after accounting for differences in plant height) needs to be optimized for different crops to ensure high-quality images for detecting genotypic differences. Algorithms will need to be developed and standardized to capture differences in color and area of foliage and anthers, accounting for soil surface in crops such as wheat where the canopy does not close completely.

### Photosynthetic efficiency

Designing ideotypes to maintain improved productivity under heat and drought stress and moving beyond stay-green versus senescence concepts, implemented at the plant level and small plots to phenotype diversity panels on large area, has been the major bottleneck. Progress achieved in sensor technology provides the vehicle to capture temporal (flowering till maturity) changes in stay-green versus senescence patterns that will allow for capturing the diversity required to incorporate into breeding programs. Experiments involving large diversity panels will need to be designed innovatively to be able to capture the gradient of changes in stay-green and senescence both within and between genotypes.

### Tracking water-soluble carbohydrate translocation to grains

Limited progress has been achieved in employing sensor-based technology to capture the storage and translocation of WSCs in plants because of the dynamic changes, both spatially (leaf, stem, and grain) and temporally (within and between days during grain filling). This is further complicated by the stage, duration, and intensity of stress which warrant the need to capture the dynamics but still establish a practically feasible approach. Taking sensor-based carbon balance in different plant parts in the morning and evening throughout the grain-filling period could help establish solid benchmarks. Using these established benchmarks, environment-specific temporal intervals (in days) can be defined at which images need to be taken that are both practical and capture >90% of changes between flowering and physiological maturity. Further, the community would still need to improve the accuracy of capturing the changes in WSCs in plant parts, building on the progress achieved by Dreccer *et al.* (2014) and Garriga *et al.* (2017).

### Estimating grain number and weight under stress

Heat and drought stresses during flowering and post-flowering stages induce non-uniform seed set (gaps) within panicles and heads, which deviates from the normal fully filled panicles that the system (Fig. 3A, B) has been optimized to estimate. Having a mosaic of loss in seeds within panicles due to stress will challenge the approach developed. This would require extensive training before it can be employed or used effectively to estimate the seed loss under stress. Currently, the integration of machine learning tools into the approach could help but would still require a large sample size with different proportions of loss in seed numbers in panicles or heads before the technology can be standardized. Unlike loss in seed numbers, reduction in seed weight within panicles and different genotypes due to heat and drought stress presents a lesser challenge and can be captured using the current model.

## Conclusions

The review provides an overview of current advances and future directions of key physiological processes related to heat and drought stress resilience during reproductive and grain-filling periods. In order to take advantage of naturally occurring trait variation to increase heat and drought stress resilience in crop varieties, collaborative science is imperative and inevitable. Tools in machine and deep learning in relation to agriculture are becoming fundamentally critical for evaluation of these hard to quantify and time-sensitive traits. In order to make progress at the rate which is required by global demand in a changing climate, traditional and hand measurements must be evolved in order to accurately, quickly, and reliably obtain more scalable measurements with high-resolution. The limitations and future research directions highlighted for the four key areas provide the next steps to establish high-throughput phenotyping platforms for field-based estimations and for incorporating these traits into global abiotic stress breeding programs.

## Abbreviations

CNN: convolutional neural network
LIFT: laser induced fluorescence transient
NDVI: normalized difference vegetation index
NIRS: near-infrared spectroscopy
PLSR: partial least squares regression
PRI: photochemical reflectance index
TOF: time of day of flowering
UAV: unmanned aerial vehicle
QY: quantum yield
WSC: water-soluble carbohydrate
